# Effect of a telecare-based intervention on stress levels in informal caregivers of older adults: protocol for a randomized controlled trial

**DOI:** 10.3389/fpsyt.2023.1167479

**Published:** 2023-05-25

**Authors:** Arkers Kwan Ching Wong, Nga Ping Ng, Vivian Chi Ching Hui, Jed Montayre

**Affiliations:** School of Nursing, The Hong Kong Polytechnic University, Kowloon, Hong Kong SAR, China

**Keywords:** caregiving, telenursing, community, older people, protocol

## Abstract

**Significance:**

Due to caregiving commitments, caregivers of older adults may not have the time to make use of the onsite community services available to them during the day. With the support of advanced technology, telecare could be a convenient and easily accessible channel for providing individualized caregiving advice to caregivers.

**Objective:**

The aim of the study is to describe a research protocol that highlights the development of a telecare-based intervention program for reducing stress levels in informal caregivers of community-dwelling older adults.

**Methods:**

It is a randomized controlled trial. The study is supported by two community centers. The study participants will be randomly assigned to either the telecare-based intervention group or the control group. The former will receive a 3-month program comprised of three components: online nurse case management supported by a health and social care team, an online resource center, and a discussion forum. The latter will receive the usual services that provided by the community centers. Data will be collected at two time points – pre-intervention (T1) and post-intervention (T2). The primary outcome is stress levels, while secondary outcomes include self-efficacy, depression levels, quality of life, and caregiving burden.

**Discussion:**

Besides taking care of one or more older adults, informal caregivers have to deal with work, chores, and take care of their children. This study will add valuable information to the knowledge gap on whether telecare-based interventions with the support of an integrated health-social team can alleviate the stress levels of informal caregivers of community-dwelling older adults. If successful, policymakers and healthcare professionals should consider incorporating telecare modalities in a primary health setting for informal caregivers to correspond with them, to relieve their caregiving stress and promote a healthy life.

**Clinical trial registration:**

https://www.clinicaltrials.gov/, NCT05636982.

## Introduction

Aging populations have become a global trend. By 2050, there will be nearly 2.1 billion people over the age of 60 – double the figure in 2017 (1). The continuously growing number of older adults will inevitably lead to a greater demand for home-based care and health-related services. In Asian countries and regions like Hong Kong, many older adults are receiving daily care from family members (i.e., 37.3% from their adult children and 26.3% from their spouse) (2). Despite their lack of caregiving skills and knowledge, research has shown that informal caregivers play a better caregiving role than do paid healthcare providers due to their strong affiliation with their older family members (3). A recent study showed a more than 20% reduction in hospital readmission rates for older adults when informal caregivers were involved during the process of planning their discharge care (4). A business survey from the United States estimated that the economic value of family caregiving for their unpaid contributions was approximately $470 billion in 2017, which greatly exceeds the value of paid home care (5). Despite these benefits to older adults, healthcare providers, and the society, the increasing reliance on informal caregivers to provide daily care to older adults may eventually place a great deal of burden and stress on the caregivers, leading to physical and emotional distress and great frustration.

An informal caregiver, which refers to an extended family member or friend who volunteers to take care of an older adult at home, usually handles different caregiving tasks in daily life, such as providing personal care, preparing meals, giving medications, and monitoring health conditions. In a qualitative study, the majority of caregivers reported while that caregiving is rewarding and their relationship with their older relative benefits from the caregiving experience, their busy caregiving role has jeopardized their personal life and commitments. For those with a full-time job and children to care for, the huge demand of providing caregiving duties to an older family member has been known to negatively affect their physical, social, financial, and emotional health. A recent survey reported that more than 60% of informal caregivers perceived that they had a heavy caregiving burden, 55% had depressive symptoms, 40% had poor family function, and 27% of them reported poor self-rated health (6). The emotional and physical demands involved in caregiving have a negative impact on the resilience of caregivers. That is why researchers and policy makers around the world have attempted to adopt different strategies to maintain and improve the health of informal caregivers and help them to perform caregiving roles.

Unlike the United Kingdom and the United States where there are structured health-social partnership frameworks, Hong Kong does not have an explicit cross-sectoral mechanism in place that facilitates information sharing among various health and social care sectors, resulting in the redundant and fragmented provision of services to both older adults and their informal caregivers (7, 8). It has been pointed out that there may be a range of different supporting services in the community available to caregivers, but they find it challenging to access the fragmented system to obtain the needed resources (9, 10).

With the rapid development of health information technology, people can now access health and social care services at home with ease. Telecare is defined as a remote intervention that allows healthcare professionals to assess the health status of clients, and provide education or deliver health and social care to them through information, communication, and monitoring technologies (11). Telecare is particularly useful to informal caregivers who find it challenging to meet healthcare professionals onsite and who have had difficulties in receiving caregiving advice due to full-time work commitments. Telecare not only allows informal caregivers to obtain caregiving information at their own time and pace, but also provides a virtual communication channel for informal caregivers and healthcare professionals from multiple disciplines to exchange caregiving ideas, seek health and social advice for their loved one, and receive physical and emotional support for their own health. Due to its many advantages, a US-based study found that more than 90% of informal caregivers were willing to participate in telecare programs that could facilitate their caregiving work and reduce their caregiving burden (12).

While studies have started to test the effectiveness of telecare on the health of older adults with multimorbidity (13), as well as on the health of young adults (14), to the best of our knowledge, the research has yet to extend to informal caregivers. Therefore, an attempt is made in this study to develop a telecare program, with the integrated efforts of a health-social team, to reduce the stress levels and caregiving burden, and improve the self-efficacy, depression levels, and quality of life of informal caregivers of community-dwelling older adults, and submit that program to empirical testing. If proven successful, policymakers and healthcare professionals should consider incorporating telecare modalities in primary health settings to correspond with informal caregivers to relieve their caregiving stress and promote a healthy life.

## Conceptual framework

Lazarus and Folkman’s Transactional Theory of Stress and Coping (TTSC) and the Creativity, Optimism, Planning and Expert Information (COPE) model will be used to guide the development of components of the intervention (15). The TTSC defines stress as resulting from an imbalance between environmental demands and individual resources. When applied to the informal caregivers of older adults, the environmental demands could include inadequate caregiving knowledge and skills, a lack of time to search for available supporting services, and inadequate physical and emotional care support; while individual resources may comprise education from healthcare professionals on caregiving knowledge and skills, community services for caregivers, and support from peers, friends, and family. To overcome the imbalance, the theory suggests two broad categories of coping mechanisms: problem-focused coping and emotion-focused coping. The aim of problem-focused coping is to manage the causes of the stressors, while emotion-focused coping is about managing the emotional distress caused by the stressors. Effective coping can lead to positive outcomes such as a reduction in the stress and depression levels of caregivers.

**Figure 1:**
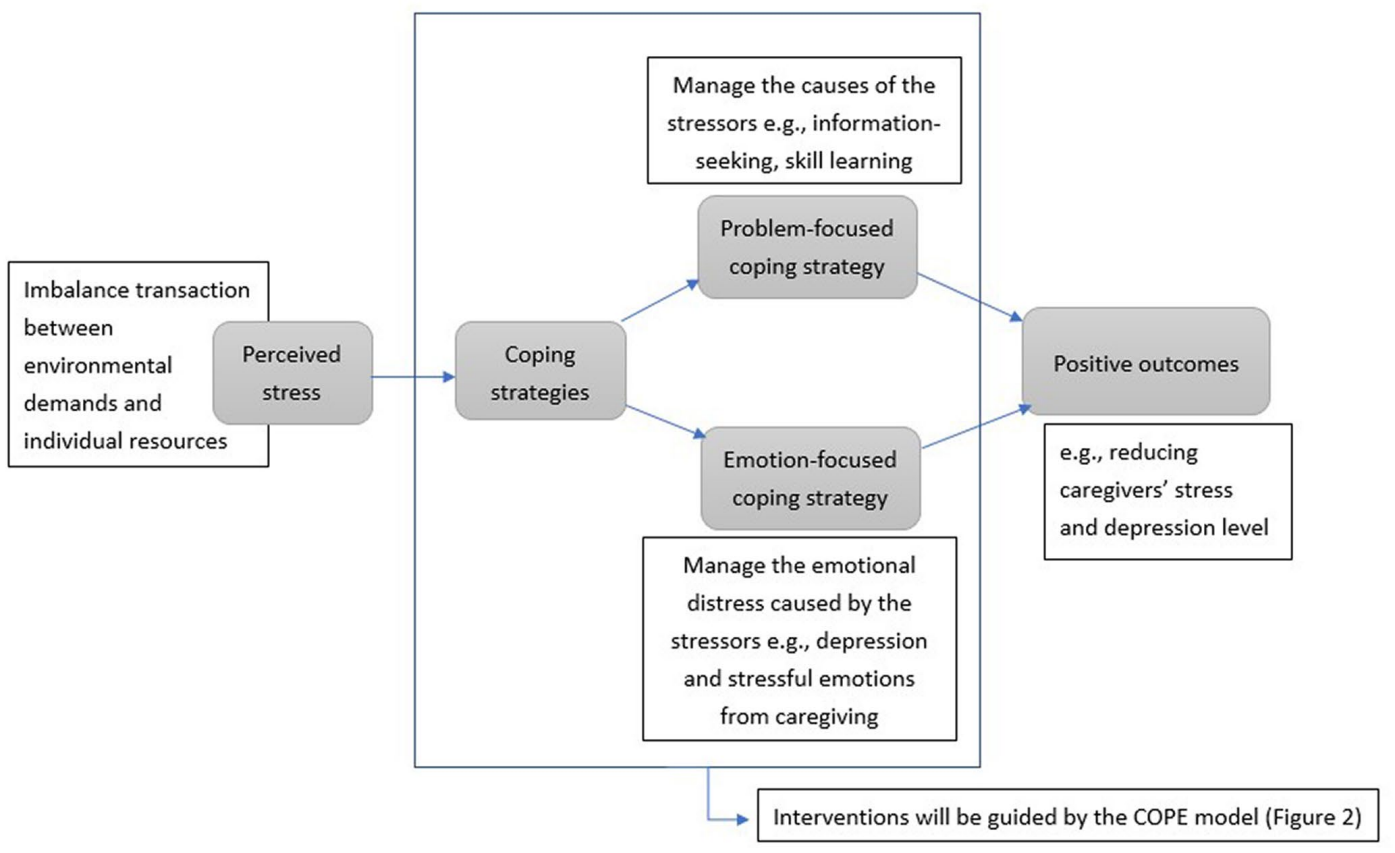
Lazarus and Folkman’s Transactional Theory of Stress and Coping.

**Figure 2:**
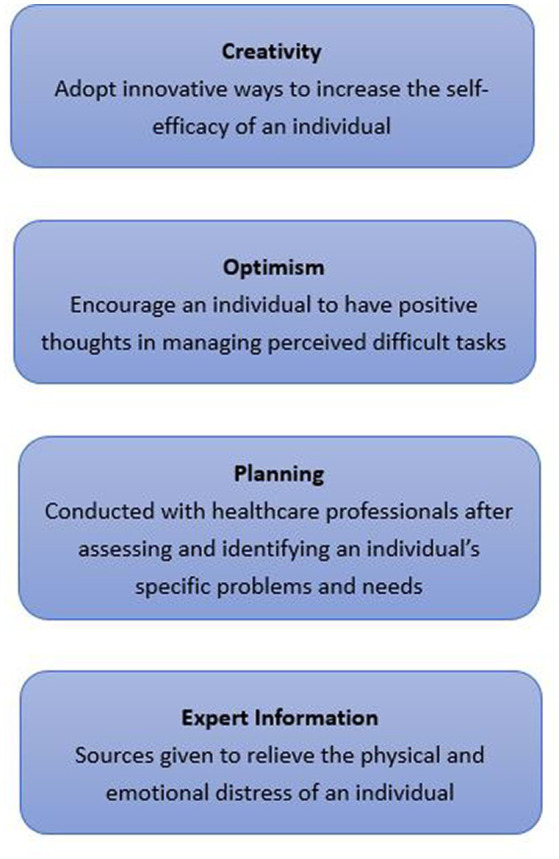
The COPE model.

The COPE model will be used to guide the development of both problem-focused and emotion-focused coping strategies (16). *Creativity* refers to the adoption of innovative ways to increase the self-efficacy of an individual. *Optimism* encourages an individual to have positive thoughts about managing tasks perceived to be difficult. *Planning* is conducted with healthcare professionals after they assess and identify an individual’s specific problems and needs. *Expert Information* will be given to relieve the physical and emotional distress of an individual. Figures 1, 2 illustrate the framework.

## Methods

The SPIRIT statement was used as a guideline for this protocol paper (17).

### Aim

The aim of the study is to evaluate the effectiveness of a telecare-based intervention on stress levels in informal caregivers of older adults.

### Study design and setting

This is a single-blinded, two-armed, randomized controlled trial, where the research assistant who is responsible for data collection is blinded but the provider is not blinded. This study is supported by two community centers of a non-governmental organization.

### Participants and recruitment strategy and randomization

A trained community center staff member will help to generate a list of potential subjects from their membership list. Potential subjects will be called and recruited into the program if they fulfill the following inclusion criteria: (1) aged ≥18 years old, (2) have no cognitive impairment as evidenced by a score of 22 or above in the Hong Kong version of the Montreal Cognitive Assessment (18), (3) can understand and communicate in Cantonese, (4) provide care to older adults aged ≥60 at least 4 h per week for a minimum of 3 months, (5) use a smartphone, (6) know how to access the Internet, (7) commit to attending bi-weekly, 15–30 min online meetings with the program providers over the 3-month period, and (8) are willing to receive individual-specific video messages covering caregiving skills via WhatsApp. They will be excluded if they are (1) known to have serious alcohol and drug problems, (2) are unable to write and read, (3) have mental health problems, and (4) have already participated in other telecare health or social programs. When two or more potential subjects are taking care of the same older adult, only the one who has spent more time with the older adults is allowed to participate.

A trained research assistant (RA) will introduce the program and invite eligible subjects to join the program in the community centers, and obtain verbal consent upon their agreement. After collecting their baseline data, the research assistant will call the principal investigator (PI) of the research team for randomization. The PI will put the numbers generated from a software program called “Research Randomizer” into sealed envelopes (i.e., “1” = intervention group, “2” = control group). These envelopes will be opened sequentially when a call is received from the RA. In this study, the RA and the community center staff will be blinded, but the PI and the healthcare providers will not.

### Interventions

This study is a 3-month program involving two parallel groups.

### Intervention group

Each intervention group subject will receive three intervention components: (1) online nurse case management supported by a health-social partnership team, (2) individual-specific video messages covering caregiving skills sent via WhatsApp, and (3) an online information center and discussion forum via a password-protected, newly-developed caregiver website.

1. Online nurse case management supported by a health-social partnership team

Each intervention group subject will be assigned to a nurse case manager (NCM) during the first week of the program. The NCM has extensive experience in providing care to informal caregivers and has been trained specifically for this program. She will meet with the subjects bi-weekly via Zoom. The time of the meetings will be settled with the agreement of both the NCM and the subjects. In the first Zoom consultation, the NCM will assess the caregiving needs and problems of the subjects by using a structured and validated assessment-intervention-evaluation tool: The Omaha System. The Omaha System consists of 42 problems under four domains ─ environmental, psychological, physiological, and health-related behaviors (19). The NCM will empower the subjects to set goals and develop a personalized care plan tailored to their identified caregiving needs and problems. The goals and care plan will be followed up and revised in subsequent Zoom consultation sessions. The NCM will also provide emotional support, encourage the subjects to discuss their concerns, identify perceived challenges to achieving their goals, and together with the subjects find solutions in the Zoom consultation sessions on how to tackle these barriers. When deemed necessary, based on a jointly developed protocol and the problems of the subjects, the NCM will refer the subjects to one of the health-social partnership team members, which includes social workers, traditional Chinese medicine practitioners, a physiotherapist, and a mental health nurse. The protocol indicates the roles and responsibilities of each member in the health and social team, the suggested assessment, interventions and an evaluation scheme for each member, and the arrangement and logistics of the program. A monthly conference will also be held among the team members to review the referred subjects’ condition, care plans, and goal status.

1. Individual-specific video messages covering caregiving skills sent via WhatsApp

In addition to online case management sessions, the NCM will send weekly, individual-specific videos of tips and reminders to the informal caregivers via WhatsApp, an interactive communication application. The subjects will receive videos that include, but are not limited to, the topics of common chronic diseases and their management, caregiving skills and techniques, available community services and resources, and self-care strategies. All of the videos originate from reliable organizations such as the Hospital Authority, Department of Health, and The Hong Kong Council of Social Service. To ensure the subject understands the content of the videos, the NCM will also teach and explain the content of the video, and answer any questions that they have during the online consultation sessions.

1. Online information center and discussion forum accessed via a password-protected, newly-developed caregiver website

The research team will work with a technology company to design a website that incorporates two features, an online information center and a discussion forum. In the online information center, the members of the health-social team will regularly post general information to the informal caregivers that have been proven to be effective for them, such as family relationship management strategies, stress relieving techniques, and physical and mental health protection skills. In the discussion forum, the subjects can raise questions, either in the form of text, video, or both text and video to the health-social team members. To answer the questions, the team members will browse the discussion forum every 3 days at minimum. The forum can also serve as a peer-support platform where all of the caregivers in the intervention group can share their individual caring experiences, discuss commonly encountered issues, and seek emotional support from their peers. Peers with a similar background and experiences are believed to have a better understanding of their feelings and to be able to provide support to and receive consolation from each other. The website is only accessible to the health social team members and members of the intervention group, who will be given a preset password and username to log in. The subjects will also be told not to disclose the password to the others and all the information posted can be viewed by the people who is allowed to access the website.

### Control group

Both intervention and control group participants will receive services from the community centers as usual. The community centers will provide regular onsite educational sessions and workshops for enhancing the caregiving knowledge of caregivers. Attendance at these lectures is not mandatory, and all subjects are welcomed to join.

### Data collection

Data will be collected at two time points – pre-intervention (T1) and post-intervention (T2). The data will be collected through phone call by a trained RA. Five percent of completed questionnaires (i.e., 4) will be randomly selected for an independent review to ensure the quality of the collected data.

## Outcome measures

### Primary outcome

The primary outcome of the study is the stress level of the caregivers. Stress levels will be measured using the 14-item Chinese version of the Perceived Stress Scale (PSS) (20). It has been applied extensively to measure the degree of perceived stress of informal caregivers. The total score ranges between 0 (minimum stress perceived) and 56 (maximum stress perceived), with higher scores representing greater perceived stress levels. The Chinese version was shown to have good validity and reliability. The Cronbach’s alphas of the total scale and the subscales were 0.881, and 0.793, 0.892 (21).

### Secondary outcomes

The secondary outcomes include self-efficacy, depression levels, quality of life, and caregiving burden.

*Self-efficacy* – The General Self-Efficacy Scale (CGSE) will be used to assess the self-perceived ability to cope with stressful life events (22). The Chinese version of CGSE contains 10 items and is scored on a 4-point Likert scale ranging from 1 (not at all true) to 4 (exactly true). The total score ranges from 10 to 40, with higher scores indicating a greater belief in one’s competence to deal with the difficulties. High internal consistencies were found, with a Cronbach’s alpha of 0.91.

*Depression* – Depression levels will be measured using the Chinese version of The Center for Epidemiologic Studies Depression Scale (CES-D). The CES-D is a self-reported scale that is used to measure the presence of depressive symptoms (23). This 4-point scale is a 20-item instrument. Total scores range from 0 to 60, with higher scores indicating more depressive symptoms. The scale was found to have a sensitivity and specificity of 100 and 76%, respectively, for a cut-off score of 16. The Cronbach’s alpha coefficient was 0.86.

*Quality of life* – The Chinese (HK) version (SF-12v2-HK) of the 12-item Short Form Health Survey version 2 will be used to assess the quality of life of the informal caregivers. The questionnaire contains 12 items involving different domains of health concepts, which include physical functioning, role limitations due to emotional problems, role limitations due to physical problems, a mental health scale, general health, bodily pain, and social functioning and vitality (energy/fatigue) (24). The internal consistency (Cronbach’s alpha) of this scale is >0.7 and its test–retest reliability estimates (ICC) is >0.7.

*Caregiving burden*– Caregiving burden will be measured using the Zarit Burden Interview (ZBI) (25). It contains 22 items and is scored on a 5-point Likert scale ranging from 0 (never) to 4 (nearly always). The total score ranges from 0 to 88, with a score of 24 or above possibly indicating a higher chance of developing depression. The sensitivity and specificity of the ZBI were 85.2 and 74.8%, respectively, for a cut-off point of 34. The measure has been widely used in studies focusing on informal caregivers, and the Chinese version of the ZBI is highly reliable and valid (26).

### Background demographic data

**Figure 3:**
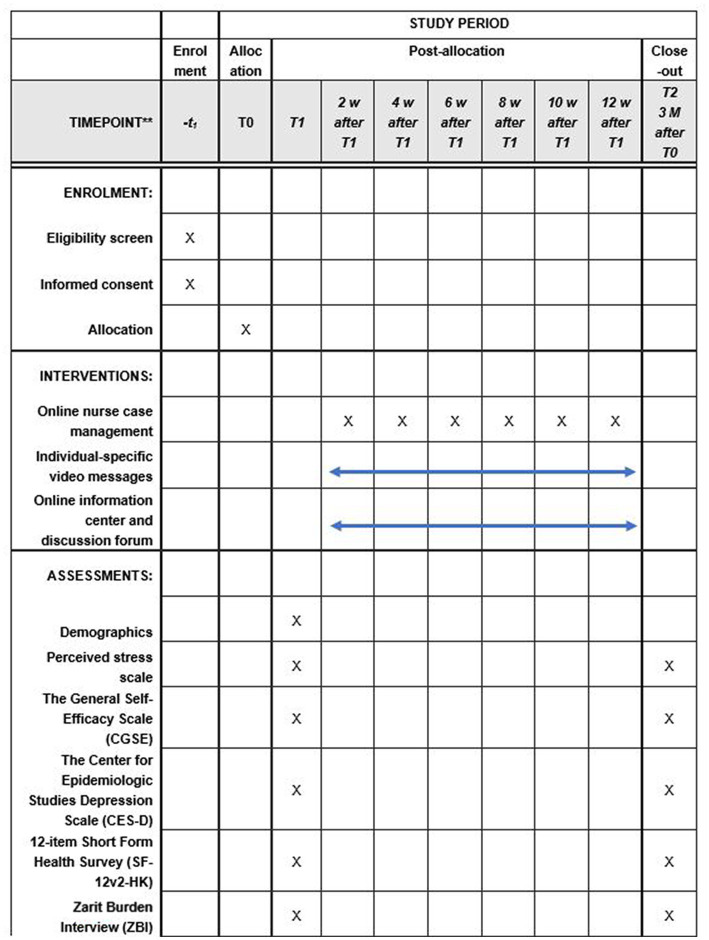
Schedule of enrollment, interventions, and assessments.

The background demographic data of the participants will be collected at baseline (T1). The data will include age, gender, marital status, level of education, number of caregiving hours per week, work status, duration of caregiving, financial situation, family structure, person with whom they live, and experience of using a smartphone. See Figure 3.

### Ethical considerations

Ethical approval was obtained from the Ethics Sub-committee of the study university. The RA will convey information about the program, including its potential benefits, risks, and the issue of confidentiality during subject recruitment. Each participant who agrees to participate in the program will be required to sign a consent form upon enrollment. The collected data will be accessed solely by the research assistants and no personal information will be disclosed. Such data will include the demographic data of the subjects, the outcome measures, and the user ID and password for accessing the virtual discussion forum. All data will be stored in a password-protected database.

### Sample size

The sample size will be calculated based on a power analysis. Assuming a two-tailed alpha of 0.05, a probability of 0.2 for the beta error (80% power), and an effect size of 0.403 after referring to a recent telephone-based mHealth program with the same primary outcome measure (perceived stress level) (27), 34 people per group will be required. With reference to the 10–15% attrition rate reported in the previous program (28), we assume a 15% drop-out rate in this study. Thus, the total sample size required is 80 participants (i.e., 40 subjects per group).

## Data processing and analysis

The Statistical Package for the Social Sciences (SPSS) version 26.0 will be used to conduct a statistical analysis. The demographic data of the intervention and control groups will be compared at baseline, using appropriate tests (i.e., Mann–Whitney U, *t*-tests, or chi-square). Depending on the normality of the data, an independent *t*-test or a Mann–Whitney *U* test will be used to determine the group differences in both primary and secondary outcomes in T2. Intention-to-treat will be employed as the primary analysis in this study. Potential covariates, such as the duration of caregiving, the number of caregiving hours per week, and the number of referrals will be examined. Within-group differences will be analyzed using either the Wilcoxon signed-rank test (non-parametric) or a paired t-test (parametric). A significant result is indicated when the *p*-value (level of significance) is less than 0.05 for a two-tailed test.

### Validity and reliability

This study uses a rigorous research design with a representative and predetermined sample. The chosen instruments were validated with high reliability and validity. In addition, the data collectors and the participants were blinded to the intervention group assignment to reduce biases in the evaluation of the effects of the intervention.

## Discussion

The role of informal caregivers is pivotal in maintaining the ability of older adults to live independently in the community. However, the multiple health and social needs of these older adults have imposed a significant burden on informal caregivers. In previous studies, a great deal of effort was expended on investigating the needs of informal caregivers of older adults with chronic diseases such as stroke, dementia, and heart failure, although in none of the studies has the idea of finding ways to relieve their caregiving burden been put forward. As informal caregivers always make caregiving one of their top priorities, they end up sacrificing their personal time, resulting in isolation from their social circle or absence from work. The aim in this study is to take advantage of technology to provide telecare consultations aimed at relieving the stress levels and caring burden of these unpaid informal caregivers.

The strength of this study lies in its use of technology to maximize the benefits to time-conscious informal caregivers. Telecare is an Internet-based tool that is not restricted by the limitations of time and place. An online information center in this program allows caregivers to access the information that they need at their convenience and to decide when and where to read about the available sources. The informal caregivers do not need to join onsite community services, so they can save time and plan their own schedule, and come up with better arrangements to care for their loved one. With the implementation of telecare, informal caregivers can enjoy supporting services while staying at home.

In addition to the use of telecare, the support of the health-social partnership team is another strength of this study. Those in health-social partnerships work together for the benefit of the clients through effective communication and collaboration. A health-social team brings together healthcare knowledge and suggestions from people with expertise in different fields, so that different aspects of needs such as the physical, mental, and social needs of the informal caregivers can be covered. Operating a health-social team is not easy because good collaboration with the shared goals of different healthcare providers is required, as well as effective exchanges of information between them. The health-social partnerships may encounter various barriers, such as complex intersectoral collaboration, and a lack of shared values and effective communication (29). A great deal of effort should be put into ensuring that the health-social partnerships work in an orderly and efficient way. In view of this, the research team has developed protocols with the health-social team to guide the work of each healthcare provider so that they have an idea of what each other’s roles and responsibilities are. They will also hold regular meetings to discuss the needs of caregivers and the progress of the case, so that they can provide true interdisciplinary care to the informal caregivers.

Telecare would not only be beneficial to informal caregivers, but also to older adults and to society as a whole. Telecare enables caregivers to be well equipped with caregiving skills and knowledge, without the restrictions of place and time. This will ultimately help their care recipients to maintain their health and reduce the need to be admitted to a nursing home and/or hospital. As a result, the healthcare system can be less overstretched and healthcare costs can be brought down with decreased health service utilization among community-dwelling older adults.

### Limitations

Although this study has various strengths, there will be some challenges in its implementation. One possible challenge could be that the informal caregivers do not regularly visit the website or actively participate in the online discussion forum. To tackle this issue, every week the NCM will check the browsing data of the website and encourage the related participants to visit the website during a Zoom meeting. Second, the participants may have technical problems accessing the website or discussion forum. The research team will provide IT support to those caregivers who face such difficulties during the program period. Third, those caregivers who are not able to read or write may not be benefited from some parts of the intervention program, such as online information center and discussion forum. However, they can still enjoy the conversation with the NCM and the NCM will try to provide them the caregiving skills and techniques through sending weekly videos.

## Conclusion

Telecare seems to be a feasible approach to providing support to the informal caregivers of community-dwelling older adults. With the support of a health-social partnership team, it is expected that the benefits of telecare can be maximized. It is anticipated that this program, substantiated by evidence, can be implemented and sustained in the community to provide long-lasting support to both older adults and their informal caregivers.

## Ethics statement

The studies involving human participants were reviewed and approved by the Research Committee of the Hong Kong Polytechnic University (reference no.: HSEARS20220810001). The patients/participants provided their written informed consent to participate in this study.

## Author contributions

AW and NN developed the conception and design of the initial study. AW was responsible for obtaining funding. AW, NN, VH, and JM drafted, revised, and approved the manuscript. All authors contributed to the article and approved the submitted version.

## Funding

This work was supported by a grant from the Undergraduate Research and Innovation Scheme (URIS) of the Hong Kong Polytechnic University (Ref No. 1-TA46). The funder had no role in the study design, data collection, analysis, decision to publish, or preparation of the manuscript.

## Conflict of interest

The authors declare that the research was conducted in the absence of any commercial or financial relationships that could be construed as a potential conflict of interest.

## Publisher’s note

All claims expressed in this article are solely those of the authors and do not necessarily represent those of their affiliated organizations, or those of the publisher, the editors and the reviewers. Any product that may be evaluated in this article, or claim that may be made by its manufacturer, is not guaranteed or endorsed by the publisher.
